# Comparison of Phase-Based 3D Near-Field Source Localization Techniques for UHF RFID

**DOI:** 10.3390/s16070978

**Published:** 2016-06-25

**Authors:** Andreas Parr, Robert Miesen, Martin Vossiek

**Affiliations:** Institute of Microwaves and Photonics, Friedrich-Alexander-Universität Erlangen-Nürnberg, Erlangen 91058, Germany; robert.miesen@fau.de; (R.M.) martin.vossiek@fau.de (M.V.)

**Keywords:** MIMO radar, phased arrays, planar arrays, radiofrequency identification, spatial filters, synthetic aperture radar

## Abstract

In this paper, we present multiple techniques for phase-based narrowband backscatter tag localization in three-dimensional space with planar antenna arrays or synthetic apertures. Beamformer and MUSIC localization algorithms, known from near-field source localization and direction-of-arrival estimation, are applied to the 3D backscatter scenario and their performance in terms of localization accuracy is evaluated. We discuss the impact of different transceiver modes known from the literature, which evaluate different send and receive antenna path combinations for a single localization, as in multiple input multiple output (MIMO) systems. Furthermore, we propose a new Singledimensional-MIMO (S-MIMO) transceiver mode, which is especially suited for use with mobile robot systems. Monte-Carlo simulations based on a realistic multipath error model ensure spatial correlation of the simulated signals, and serve to critically appraise the accuracies of the different localization approaches. A synthetic uniform rectangular array created by a robotic arm is used to evaluate selected localization techniques. We use an Ultra High Frequency (UHF) Radiofrequency Identification (RFID) setup to compare measurements with the theory and simulation. The results show how a mean localization accuracy of less than 30 cm can be reached in an indoor environment. Further simulations demonstrate how the distance between aperture and tag affects the localization accuracy and how the size and grid spacing of the rectangular array need to be adapted to improve the localization accuracy down to orders of magnitude in the centimeter range, and to maximize array efficiency in terms of localization accuracy per number of elements.

## 1. Introduction

The origins of phase-based near-field source localization (NFSL) are firmly grounded in direction-of-arrival (DOA) estimation. A great number of signal processing methods, algorithms and system architectures have been developed to determine the azimuth and elevation of impinging electromagnetic waves with the lowest possible error under the most varying conditions [1]. The direction-of-arrival estimation methods use the plane-wave approximation for far-field conditions in which the distance from the receiving antenna array to the source of the wave is much greater than the array dimensions. As the antenna to source distance gets smaller versus the array size, the approximation for plane waves no longer holds and the actual shape of the spherical wave fronts has to be taken into account. Near-field source localization exploits the distance-dependent wave front shape in the near-field region of an antenna array to estimate not only the angle to a target, but its location in 3D space [2,3].

The electromagnetic (EM) waves used by a reader and a tag in a backscatter scenario or in UHF RFID, as discussed in this paper, enable NFSL for these technologies. Obtaining spatial information about RFID tags is topic of current research and several approaches that enable RFID localization have been published. Most work is published on phase- or signal-strength-based measurements for 2D localization [4]. Signal-strength-based localization approaches compare the measured RSSI at multiple antenna positions with an EM-propagation-model to estimate the tag positions [5,6], whereas phase based localization approaches evaluate the signal phase of the narrow band receive signal [7]. The localization systems use either measurements with static antenna positions as in [8,9], or measurements with moving tags [10], or moving antennas, which can be mounted on mobile robots [11,12,13].

The combination of NFSL and a backscatter technology delivers a variety of new transceiver concepts as the source does not emit a wave by itself, but is illuminated by a transmit antenna. By choosing different transmit and receive antennas that are part of an array, additional signal paths are created during one position estimation, which lead to different localization properties and transceiver complexities. Researchers in [8,9] have shown that near-field single input multiple output (SIMO) and multiple input multiple output (MIMO) localization systems that use every array element in transmit and receive mode provide much better localization accuracy and do not require a high spatial sampling rate, as is needed in far-field systems.

Despite the large body of work published on UHF RFID localization, very little work has addressed the localization of UHF RFID tags in 3D. The authors in [14] present a Received Signal Strength Indicator (RSSI) based 3D localization system that yields promising results with a four-element 3D antenna array. However, research into phase-based 3D RFID localization is very rare and the accuracies one can expect in such a system have not yet been investigated.

This paper therefore addresses multiple approaches from phase-based NFSL, DOA and UHF RFID localization and assesses their suitability for 3D UHF RFID localization with 2D antenna arrays under multipath conditions. We conduct simulations to compare the performance of different localization algorithms, transceiver modes and antenna geometries in terms of localization error and system complexity. The localization accuracy strongly depends on various array-, algorithm- and system-properties, as we will show by multiple Monte-Carlo simulations and measurements with uniform rectangular arrays (URA) in stationary tag scenarios. The spatial information determined by backscatter NFSL could be used to great value in cutting-edge localization systems in logistics, healthcare and robotics. The presented methods are especially suited for use with synthetic apertures, i.e., moving antennas. Synthetic apertures have been used for 2D phase based RFID localization before [15] and have the great advantage of efficiently deploying high array element numbers with low array element pitches.

The main contributions of this paper are:

1. We introduce a simulation model that is capable of evaluating phase based narrowband backscatter localization systems. We present a new and beneficial approach in which we numerically analyze localization accuracies for UHF RFID with the aid of a realistic multipath model incorporating spatial signal correlation
2. We evaluate and compare three known and one novel backscatter transceiver modes and five backscatter localization algorithms (two of these algorithms are our own adaptions) with respect to their 3D localization accuracy under multipath conditions.
3. We show how the localization error depends on the distance between array and tag. We assess different array sizes and URA grid spacings with respect to their 3D localization accuracy and show which geometries deliver the best and most efficient results in terms of element number per localization accuracy.
4. The comprehensive analysis enables an empirical and yet sophisticated insight into the impact of system parameters on localization accuracy in phase-based UHF RFID 3D localization systems.

The paper is organized as follows. Section 2 provides the basic definitions of the array geometry and the signal model used. Section 3 introduces multiple localization algorithms. Section 4 gives a short overview of theoretical localization properties of different array shapes. Section 5 introduces multiple transceiver modes. Section 6 describes the simulation and multipath model used. Section 7 presents simulation results on the transceiver modes and localization algorithms. Section 8 presents the measurement results. Section 9 presents additional simulations that involve array geometry and distance dependency of the localization error. Section 10 draws a conclusion.

## 2. Basics

This section discusses the basic principles and definitions required for phase-based 3D backscatter NFSL.

### 2.1. Array and Geometry Definition

Consider an antenna array of *N* antenna elements with the *n*-th antenna located at position ${\vec{r}}_{a,n}$ in a 3D Cartesian coordinate system. A single tag is located at position ${\vec{r}}_{t}$, which yields all possible antenna-to-tag distances

(1) $$d_{at,n}=\left\|{\vec{r}}_{a,n}-{\vec{r}}_{t}\right\|$$

For simplification, we assume all antenna patterns to be isotropic in phase and gain. An angle dependent phase shift introduced by a real antenna can be calibrated in a real setup. However, investigations with the patch antennas used in our measurements show that the introduced error can be neglected in our measurements, as it is much smaller than the multipath induced phase error [10] and because most localization algorithms ignore a mean phase offset introduced by the antenna. The angle and polarization-dependent amplitude antenna gain has no direct influence on the proposed methods. However, additional angle and tag-orientation dependent phase shifts can be introduced which impair the localization accuracy if neglected [9], especially if circular polarized antennas are used.

### 2.2. UHF RFID Phase

**Figure 1:**
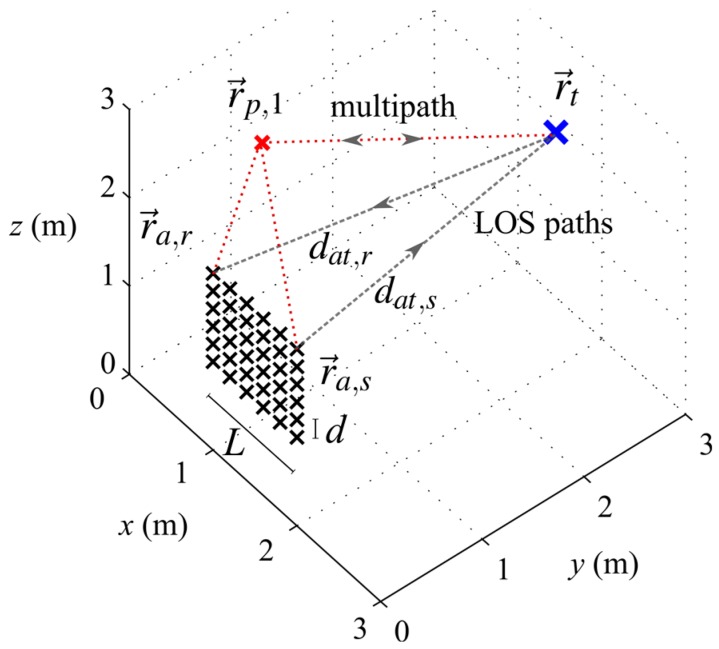
Localization setup geometry with a uniform rectangular array (URA). The line-of-sight (LOS) paths from send and receive antennas to a backscatter tag and the multipaths due to a point scatterer are depicted.

The communication between RFID reader and RFID tag yields complex receive signals. The distance-dependent phase of the modulated backscatter signal can be assigned to a single tag with a unique ID. Multiple overlapping signals therefore play a minor role for localization in RFID NFSL. Each transmit and receive antenna combination generates an individual path when used to communicate with the tag. The phase of the *i*-th measurement path is modeled as a function of the antenna-to-tag distances for the transmit and receive antennas with indices *s* and *r*, respectively:

(2) $$\varphi_{i}=\arg\left(e^{jk\left(d_{at,s}+d_{at,r}\right)}\right)+\varphi_{x,i}\;$$

where $k=2\pi /\lambda_{c}$ denotes the wavenumber of the carrier with wavelength $\lambda_{c}$ which is about 33 cm for UHF RFID and $\varphi_{x,i}$ represents unknown geometry independent phase shifts in the *i*-th path caused by the transceiver, cables, antennas or tag. Figure 1 shows an example geometry of the localization setup described above and comprising transmit and receive antennas, which are part of a URA; a tag of interest; and the line-of-sight (LOS) paths.

### 2.3. Steering Vector and Signal Model

The array steering vector $a({\vec{r}}_{t})$ is originally defined as the phase shifts needed to steer an array’s beam to position ${\vec{r}}_{t}.$ We extend the definition to the multistatic backscatter case. The *i*-th element of the steering vector now represents the phase shift of the *i*-th path with defined transmit and receive antenna:

(3) $$a({\vec{r}}_{t})=\;{\left[e^{j\varphi_{1}},\;e^{j\varphi_{2}},\ldots \;,\;e^{j\varphi_{I}}\right]}^{T}$$

with a total of *I* paths. If the array is used to obtain a phase measurement with a single tag at position ${\vec{r}}_{t}$ for all paths and a complex transmit signal *b*, a signal data vector **x** is obtained:

(4) $$x({\vec{r}}_{t})=b\;a({\vec{r}}_{t})\circ e=b{\left[e^{j{\tilde{\varphi }}_{1}},\;e^{j{\tilde{\varphi }}_{2}},\ldots \;,\;e^{j{\tilde{\varphi }}_{I}}\right]}^{T}$$

where ${\tilde{\varphi }}_{i}$ is the measured phase value of the *i*-th path and ∘ denotes the entrywise or Schur product. The error vector **e** has complex elements with unity amplitude and unknown phase. We set the transmitted signal *b* to unity amplitude and zero phase for our analysis.

## 3. Localization Algorithms

Diverse localization algorithms are available in the NFSL and DOA literature [1,16,17]. They are designed to estimate the most likely transponder position or bearing for a given measurement setup and scanned receive signals at the array elements. The following is a brief review of key algorithms suitable for scenarios involving backscatter.

### 3.1. Conventional Beamformer

The conventional beamformer (BF) “steers” the beam of an array to a desired location ${\vec{r}}_{t}.$ The steering is done offline during signal processing by digitally applying phase shifts to all measurement paths in a way, that the signals would add up coherently to a composite signal with maximum amplitude *I* if a transponder was actually in place at the steered position. If the steering vector and the signal-data vector are given, the most likely transponder position ${\vec{r}}_{t}^{\prime }$ is estimated by maximizing a target function [1]:

(5) $${\vec{r}}_{t}^{\prime }=\underset{{\vec{r}}_{t}}{\arg\max}\left(\left|a{\left({\vec{r}}_{t}\right)}^{H}x\right|\right)$$

The beamformer can also be implemented with the sample covariance matrix **R** that is obtained from *U* samples of the signal-data vector

(6) $$R=\frac{1}{U}\sum_{u=1}^{U}x[u]x{\left[u\right]}^{H}$$

where *u* is the sample index. The beamformer output follows as

(7) $${\vec{r}}_{t}^{\prime }=\underset{{\vec{r}}_{t}}{\arg\max}\left(\frac{a{\left({\vec{r}}_{t}\right)}^{H}Ra({\vec{r}}_{t})}{a{\left({\vec{r}}_{t}\right)}^{H}a({\vec{r}}_{t})}\right)$$

### 3.2. Capon’s Beamformer

Capon’s beamformer uses remaining degrees of freedom when creating the beam to minimize the average output power of the array, while maintaining maximum output power for the position it is steered to [1]:

(8) $${\vec{r}}_{t}^{\prime }=\underset{{\vec{r}}_{t}}{\arg\max}\left(\frac{1}{a{\left({\vec{r}}_{t}\right)}^{H}R^{-1}a({\vec{r}}_{t})}\right)$$

### 3.3. MUSIC Algorithm

The MUSIC algorithm uses the eigenvectors corresponding to the *I - J* weakest eigenvalues of **R** to form the noise subspace matrix **V**. *J* is the estimated number of signals. The most likely transponder position is calculated as [1]

(9) $${\vec{r}}_{t}^{\prime }=\underset{{\vec{r}}_{t}}{\arg\max}\left(\frac{1}{a{\left({\vec{r}}_{t}\right)}^{H}VV^{H}a({\vec{r}}_{t})}\right)$$

MUSIC has advantages if multiple uncorrelated signals impinge on the receive array. The method is therefore suited for measurement setups with tag-signal collisions, as in multi-reader or multi-beam setups.

### 3.4. Calibrated Beamformer

The methods discussed up to now require solely a differential calibration that compensates the phase shift differences that occur between different paths, e.g., different cables.

Phase shifts that are common to all paths, like the phase shift introduced by the tag, do not alter the estimation result. In a measurement setup with synthetic antenna elements, where the actual antennas used are the same for all paths, no calibration is needed at all. This originates from the beamformer, which is designed to maximize signal strength at the output of an array without any constraints on the phase of the received sum signal.

In an RFID localization setup, however, it can be beneficial to calibrate all phase offsets that influence the measured phase at the receiver. This includes phase shifts associated with send and receive electronics, cables as well as reader and tag antennas and the phase introduced by the tag of interest. We therefore adapt Equation (5) to

(10) r→t′=argmaxr→t(Re{a(r→t)Hx})

By using the real part instead of the norm as in Equation (5), the value of Equation (10) is maximized only as long as the phase differences between measurement and hypothesis tend to zero at all antenna elements. This can lead to an increased localization accuracy if an absolute calibration is maintained, which, however, can be difficult in real world systems, especially if the tag properties are unknown.

### 3.5. 180° Beamformer

Commercially available readers often introduce a 180° phase ambiguity [18]. The algorithms can be adapted to that case by transforming all phase shifts to the [0°, 180°] domain by multiplying all path phases by 2, which was introduced in [10]. The adapted beamformer follows as

(11) $${\vec{r}}_{t}^{\prime }=\underset{{\vec{r}}_{t}}{\arg\max}\left\{\left|\left[{\left(a({\vec{r}}_{t})\circ a({\vec{r}}_{t})\right)}^{H}\left(x\circ x\right)\right]\right|\right\}$$

Path phase errors of 180° do not alter the localization result as the algorithm wraps them. Compared to a conventional beamformer the 180° BF changes the spatial beam pattern in a way it would do, if the signal frequency was doubled. This also leads to a penalty for the interelement spacing between two antenna elements compared to a standard BF that is required for a pattern without sidebeams. The interelement spacing needs to be reduced by a factor of approximately 2 compared to the conventional BF to produce a point spread function that has similar side lobes.

## 4. Aperture Geometry

Aperture geometry greatly influences localization accuracy. Multiple different geometries have been studied and work has been carried out to reduce the sidelobe level, the required element number and beamwidth in the far-field region [19,20,21]. For the sake of simplicity, we will limit our investigations in the following to the use of URAs.

It is known that the interelement spacing *d* of a URA has a high impact on the shape of the steered beam in the far-field. The same applies to the near-field. However, if the array is steered to the near-field, the beam transforms to a bounded volume in 3D space as opposed to expanding to an infinite range in the far-field which is used in classical DOA estimation. A backscatter that is positioned in these areas generates a high array output and can be localized by means of this spatial array output filter.

If *d* is chosen too high, sidelobes (ambiguities in localization terms) arise in the beam pattern. Localization with multi-beam patterns is very susceptible to measurement errors as well as to multipath. For far-field conditions, the interelement spacing *d* needs to be kept below λ/2 so as to generate a beam pattern that has no ambiguity issues for the non-backscatter case. We will keep the size of *d* in mind during our investigations and sometimes refer to the far-field requirement for *d*. However, we will show later that the far-field requirements do not hold for the near-field case and can be violated and still maintain an ambiguity free point spread function.

Increasing the URA edge length *L* is highly desirable as it improves accuracy in the range direction and reduces the beamwidth and hence the accuracies in azimuth and elevation [22].

## 5. Transceiver Modes

**Figure 2:**
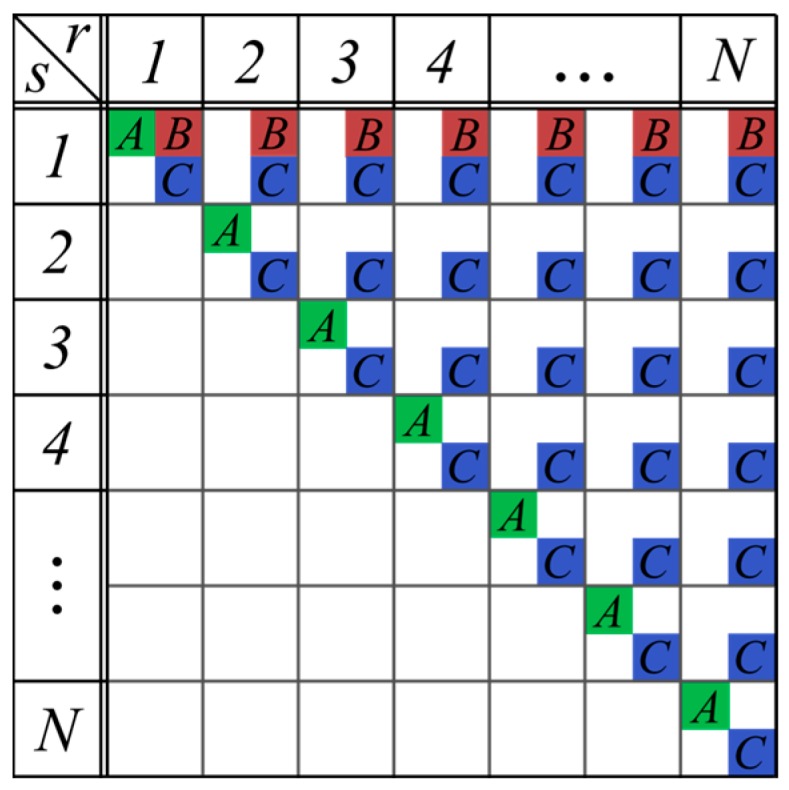
The corresponding send and receive antenna combinations with indices *s* and *r* of the transceiver modes: (**A**) Single Input Single Output (SISO); (**B**) SIMO; and (**C**) MIMO. If an antenna combination is used by a certain transceiver mode the corresponding letter is inscribed in the chart (e.g., SISO uses all antenna elements in a monostatic configuration and yields *N* paths).

The transceiver architecture and measurement procedure determines which paths can be used for the data vector **x** for a single localization. We will categorize the most important transceiver modes and discuss their properties. Figure 2 shows three fundamentally different measurement modes, denoted A, B, C, which use different send and receive antenna combinations.

### 5.1. Single Input Single Output (SISO)

The first transceiver mode uses each antenna element in the monostatic configuration. As each antenna generates one measurement path by communicating with the tag, the number of paths is

(12) $$I_{A}=N$$

As the wave travels to the tag and back, phase values increase twice as quickly with the tag distance as in the non-backscatter case. This again can be thought of as a doubled frequency compared to the non-backscatter case and leads to a required minimum interelement spacing decreased by factor 2 for a point-spread function for SISO with no ambiguities. The factor 2 again is true for far-field conditions but only holds approximately in the near-field. Still a comparatively finely spaced antenna grid is needed for high localization accuracies.

This method is similar to the holographic approach used in [15] where a single antenna is moved to generate a synthetic aperture. Alternatively, an *N*-way switch could be used with an *N*-element array and a monostatic reader.

### 5.2. Single Input Multiple Output (SIMO)

The second method uses a fixed transmit antenna element to illuminate the tag of interest [8]. All antennas act as receiving antennas and the number of bistatic measurement paths is:

(13) $$I_{B}=N$$

As the signal path to the tag is the same for all paths, this case can also be thought of as a non-backscatter case, where the tag emits a wave with a constant phase for all receive antennas. The main beam is generally broader, and there are significantly fewer sidelobes compared to the first method for the same interelement spacing. Another advantage of SIMO is that multipath in the transmit channel is the same for all paths and is ignored except by the calibrated Beamformer defined in Equation (10).

The transceiver is easily realized with a bistatic reader, and an *N*-way switch or with a stationary transmit and mobile receive antenna that generates synthetic antenna elements.

### 5.3. Multiple Input Multiple Output (MIMO)

This method uses all possible paths formed by combining all antenna elements leading to

(14) $$I_{C}=0.5(N^{2}+N)$$

unique paths [9]. The paths with interchanged send and receive antennas only yield paths that contain no additional spatial information, as the transfer function stays the same; these paths are therefore neglected. Combining all paths results in the coherent superposition of all possible versions of A and B. There are much fewer sidelobes than with other transceiver modes and the mainbeam is comparatively thin.

The method requires a complex transceiver, that is able to use each antenna in receive and transmit mode. This can be realized by a combination of multiple *N*-way switches or an *N*-way receiver as proposed in [9]. The method can be realized as a synthetic aperture by moving send and receive antennas independently to all possible position combinations.

### 5.4. Singledimensional MIMO (S-MIMO)

A new method, which we hereby propose, uses all possible MIMO paths in one dimension of a two-dimensional array, e.g., the columns in a URA. The method can be implemented easily as a synthetic aperture, e.g., by moving a uniform linear array (ULA) that has elements in *z*-direction, in *x*-direction (see Figure 1). Let us take a URA with *N* antenna elements, where each row or column has $\sqrt{N}$ elements, each column yields $0.5({\sqrt{N}}^{2}+\sqrt{N})$ unique S-MIMO paths as in Equation (14) and a total number of

(15) $$I_{D}=0.5\sqrt{N}({\sqrt{N}}^{2}+\sqrt{N})=0.5(N^{1.5}+N)$$

path is generated.

The method results in a comparatively high resolution in *x*- and *z*-directions as the advantages of the MIMO mode take effect in *z*-direction and the *x*-resolution profits from the very small antenna element distances that can be realized in *x*-direction with a synthetic aperture, while mitigating the need for an extensive number of antennas and a multidimensional synthetic aperture.

## 6. Simulation Environment

We apply simulation techniques in this work to evaluate the localization algorithms and transceiver modes introduced above with respect to their localization accuracies. Analytical approaches, like the Cramer-Rao bound, give lower boundaries for the estimators, but do not take into account all effects of ambiguities in the point-spread function or complex errors like multipath [23]. We therefore use Monte-Carlo simulations that estimate mean localization errors with the aid of a channel model that ensures realistic simulation of noise and multipath effects. The RFID channel can be modeled in general as a double fading channel, where each channel can be modeled as a Rice channel that can have a dominant LOS component [7,24]. A simple Rice channel model is normally used to estimate the amplitude’s probability density of the channel. However, when simulating a localization system, we have to ensure a spatial correlation of the channel model in amplitude and phase in order to get realistic results. If no spatial correlation is present, the mean phase errors over all antenna elements will average each other out and the estimated localization errors will be too low [25]. A channel model that ensures a spatial correlation with a superposition of signals that originate from random directions was presented in [26]. We adapt the ideas presented in [7,26] and formulate a simple but reasonable channel model. This model is based on a multipath model with *M* randomly placed point scatterers, which ensures a realistic spatial correlation of the modeled phase values.

Assume the *m*-th scatterer is located at ${\vec{r}}_{p,m}$ and the transmit antenna has index *s* and the receive antenna index *r*. We use the transfer functions for send and receive channels:

(16) $$\begin{array}{l} H_{s}=\left(e^{jkd_{at,s}}+A\sum_{m=1}^{M}\;e^{jkd_{spt,m}}\right)\\ H_{r}=\left(e^{jkd_{at,r}}+A\sum_{m=1}^{M}\;e^{jkd_{tpr,m}}\right) \end{array}$$

where *A* indicates the amplitude of each multipath signal relative to the line-of-sight (LOS) signal and

(17) $$\begin{array}{l} d_{spt,m}=\left\|{\vec{r}}_{a,s}-{\vec{r}}_{p,m}\right\|+\left\|{\vec{r}}_{p,m}-{\vec{r}}_{t}\right\|\\ d_{tpr,m}=\left\|{\vec{r}}_{t}-{\vec{r}}_{p,m}\right\|+\left\|{\vec{r}}_{p,m}-{\vec{r}}_{a,r}\right\| \end{array}$$

denote the distances the *m*-th multipath signal travels from send antenna to tag $d_{spt,m}$ and from tag to receive antenna $d_{tpr,m}$ via the scatterer. Usually the amplitude of the multipath components is Rayleigh distributed in a Rice channel. Our simulations however showed that similar results are obtained for the estimated localization accuracies if a uniform amplitude *A* is used for the multipath components. An element of the data signal vector **x** for send antenna *s* and receive antenna *r* is then emulated with

(18) $$x_{sr}[u]=b\;H_{s}H_{r}\nu [u]$$

where ν[u] is a complex noise sample with unity amplitude. We will characterize the complex noise in terms of the standard deviation of the measured phase samples, which is convenient for small noise values:

(19) $$\sigma_{\varphi }=\sqrt{\text{Var(}\arg(\nu ))}$$

As can be seen in Equation (16), a transmitted signal in the send or receive channel is the sum of the LOS signal and *M* multipath signals. The one-directional channel is now used for a multipath power definition. The mean amplitude of *M* summed complex signals with unity amplitude and random phase *X*:

(20) $$\bar{a}(M)=\bar{\left|\sum_{m=1}^{M}\;e^{jX}\right|}\;X\in [0,2\pi ]$$

was estimated numerically to yield power normalization values for the multipath signals for different values of *M*. The *K*-factor known from channel fading models can be expressed as:

(21) $$K=\frac{\text{signal power}}{\text{mean multipath signal power}}=\frac{1}{A^{2}\;\bar{a}{\left(M\right)}^{2}}$$

We will test the algorithms for varying multipath conditions defined by *K*. For *M*  > 3 the model leads to a phase error distribution very similar to Rician fading.

The simulation generates the geometrical model with antenna array and tag positions, emulates the signal data vector with Equation (18) for a given error model, runs the localization algorithms and estimates the root-mean-square (RMS) localization error

(22) $$\Delta r_{\text{RMS}}=\sqrt{\frac{1}{C}\sum_{c=1}^{C}{\left\|{\vec{r}}_{t,c}-{\vec{r}}_{t,c}^{\prime }\right\|}^{2}}$$

via *C* Monte-Carlo trials. For each trial, the transponder and scatterer positions are generated at random positions which are uniformly distributed in the target volume. We fix some of the environmental parameters to obtain comparable results. The area in which the tag is placed and searched is a cube with 3 m edge length. The number of scatterers *M* is set to 4, which leads to spatially highly correlated multipath errors. Unless otherwise stated we will use the conventional beamformer, the SIMO transceiver mode and a signal with U=100 complex signal samples with $\sigma_{\varphi }=0.2$ and no multipath. Note that it is possible to record the *U* samples during a single tag to reader communication, e.g., one sample for each data symbol. *C* is set to 500, to achieve a low standard deviation on the estimated localization errors. A 4-stage grid search with a grid spacing that starts with 5 cm in the first stage and is reduced to 5 mm in the final stage is used in the simulation and the measurements. We will use a URA aperture geometry with 1 m edge length in *x*- and *z*-direction, which is centered around the point [1.5, 0, 1.5] m for the first simulations. The interelement spacing is set to 0.2 m in *x*- and *z*-direction, which leads to 36 possible positions for send and receive antenna. Figure 1 depicts the geometrical setup.

## 7. Simulation Results I

**Figure 3:**
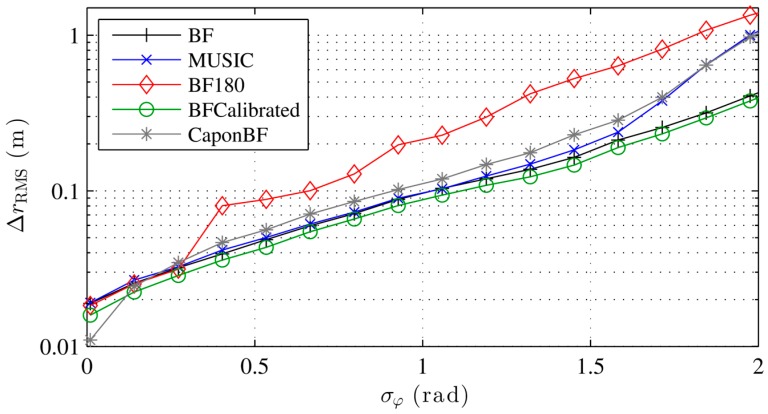
Simulated localization error with different localization algorithms for increasing phase noise errors and no multipath.

**Figure 4:**
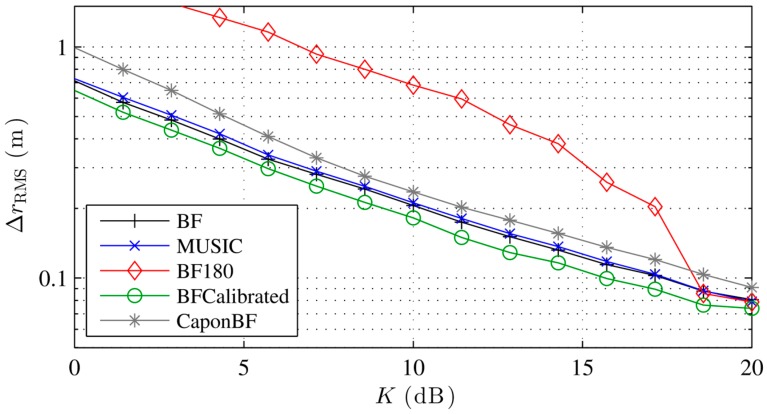
Simulated localization error with different localization algorithms for increasing LOS to multipath power and noise equivalent to $\sigma_{\varphi }=0.2.$

Figure 3 shows the localization error with different localization algorithms for an increasing phase measurement error. The conventional and Capon beamformer as well as MUSIC perform equally well in the presence of noise. The error with Capon-BF and MUSIC degrades with high noise, due to the covariance matrix implementation and the noise model described in Equation (18). While the calibrated beamformer performs slightly better the 180° BF performs poorly except with very low noise. As discussed earlier, the 180° BF requires a relatively low interelement spacing. The sudden increase in the localization error is typical for the error caused by sidelobes, which tend to maximize the beamformer output at incorrect locations more often with increasing phase error. The resulting ambiguities lead to a high error. Our simulations show that the 180° BF performs equally well as the conventional BF if the interelement distance is kept low enough, e.g., 0.05 m. Figure 4 shows similar results for a rising *K*, i.e., a decreasing multipath power.

**Figure 5:**
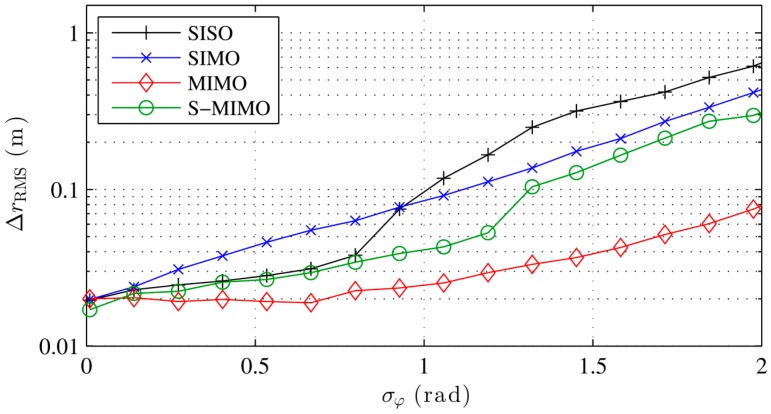
Simulated localization error with different transceiver modes for increasing phase noise errors and no multipath.

**Figure 6:**
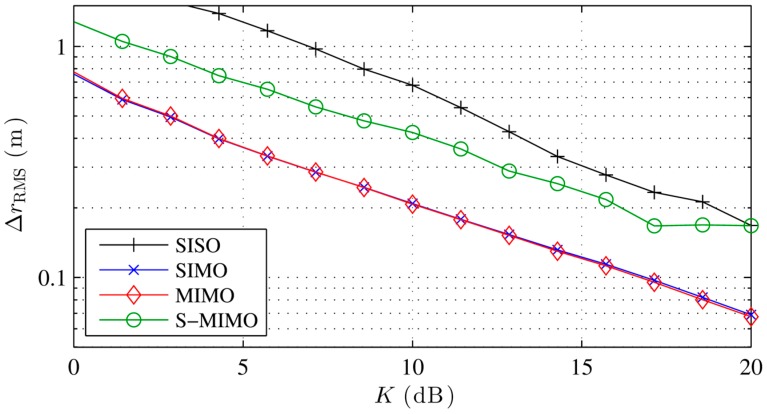
Simulated localization error with different transceiver modes for increasing LOS to multipath power and noise equivalent to $\sigma_{\varphi }=0.2.$

Figure 5 and Figure 6 show the performances of different transceiver modes for different noise and multipath. SISO performs worst if errors are present. Only in the absence of multipath has SISO a higher localization accuracy than SIMO. SIMO performs very well for multipath errors for its transceiver and measurement complexity. MIMO performs best under strong LOS conditions. However, its complex transceiver is not worth deploying in our setup, as it is not any better than SIMO at eliminating multipath issues (this is surprising as both setups are quite different). As mentioned before, SIMO has a constant multipath constellation in the transmit channel for all paths which partly explains its good performance. Apparently both methods have identical multipath mitigation properties in the used setup, which can be explained with the uniform grid, that yields no extra diversity for MIMO mode compared to SIMO. S-MIMO performs well under multipath conditions considering its simple realization.

## 8. Measurements

**Figure 7:**
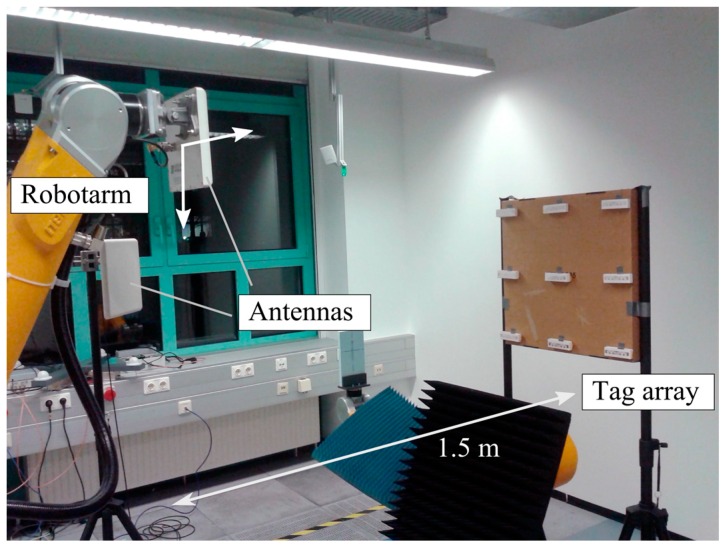
UHF RFID measurement setup with a robotic arm that generates a synthetic aperture for SISO and SIMO measurements. A tag array at 1.5 m distance is localized.

We built an exemplary measurement setup to localize 9 tags with a synthetic URA to verify theory and simulation. Figure 7 shows the measurement setup. The tags are placed at a distance of 1.5 m from the URA aperture. A robotic arm moves a single 7 dBi antenna and creates the URA. A second stationary antenna located nearby the synthetic aperture is used for bistatic SIMO links. Measurements with transceiver modes SISO and SIMO are conducted. The robot generates a URA with an interelement spacing of 8 cm, an edge length of 0.8 m and a total element number of 121. We used a 180° BF localization algorithm due to a 180° phase ambiguity that is introduced by the used reader. The localization uses a single phase measurement per path with a standard deviation of 0.01 rad. To compare the phase noise to our simulations we have to keep in mind that the simulation uses 100 samples. The measurement noise therefore matches the simulation for a standard deviation of $0.01\sqrt{U}=0.1.$

**Figure 8:**
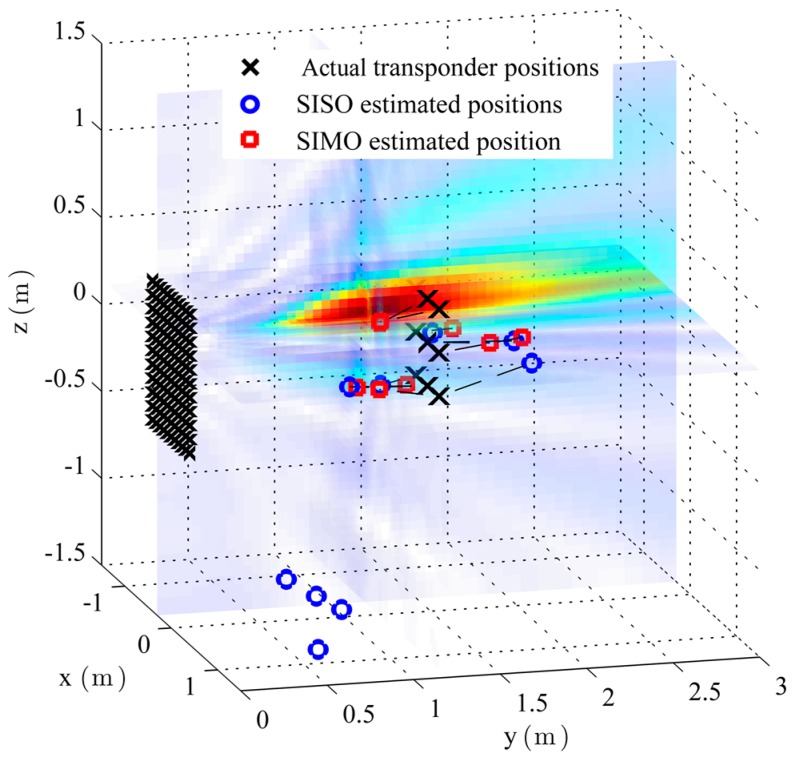
Measurement setup geometry with a 121 element URA. The actual and estimated tag positions are indicated, as well as a steered SIMO beam created by the synthetic aperture.

**Figure 9:**
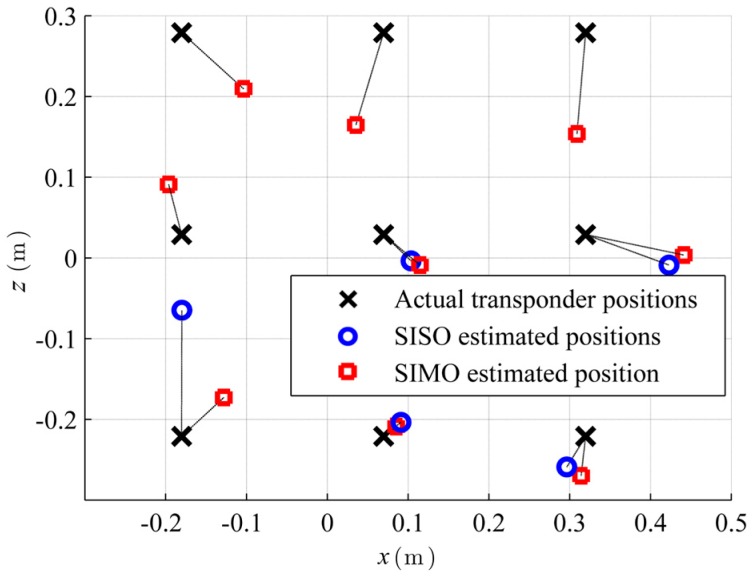
Projection of the measurement localization results in the *x*-*z*-plane. SIMO reaches an RMS error of 9 cm.

Figure 8 shows the measurement setup with antenna aperture, tag array, calculated target function heat map and with the position estimates of the SIMO and SISO measurements. SISO locates only five of the nine tags with an error less than 50 cm. Four position estimates are situated in a sidelobe that is caused by the relatively high interelement spacing in combination with the corresponding disadvantages of the SISO mode and the 180° beamformer with respect to sidelobes. The RMS error for SISO is 1.2 m. The RMS error for SIMO is 33 cm. As can be seen, the *y*-resolution suffers as the distance is relatively large compared to *L.* Figure 9 therefore shows a projection of the actual and estimated transponder positions onto the *x-z* plane. The error in the *x-z* plane reduces for SIMO to 9 cm.

To compare measurements and simulations a value for *K* is estimated for the measurement setup by comparing the mean phase error $\bar{\Delta \varphi }$ of multiple measurements with known transponder positions to our multipath model. We estimated K≈10 dB for the measurement setup from a mean multipath induced phase error of 0.4 rad per path. A comparison with the simulations shows, the error in the actual setup is strongly multipath induced. The phase error of 0.1 induces a very small localization error compared to the localization error simulated for the measured *K*.

## 9. Simulation Results II

**Figure 10:**
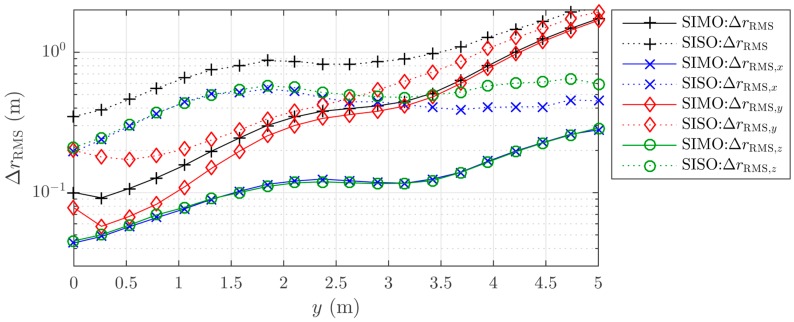
Simulation of the localization errors in *x*-, *y*- and *z*-directions with parameters that match the measurement setup (*K* = 10 dB, $\sigma_{\varphi }=0.1,$ 180° beamformer localization algorithm, and SIMO and SISO transceiver modes) for different *y*-values of the tags of interest.

Additional simulation results with parameters that match the measurement setup and localization algorithm are depicted in Figure 10. The figure shows the mean errors separately for the different coordinate axes and transceiver modes. In contrast to our prior simulations, the *y*-position of the randomly placed tags is now fixed to a certain range and the localization error is plotted with respect to that position. The simulation shows, how the localization error grows with the distance between tag and array. The *y*-error especially rises quickly and dominates the total error for distances above 2.5 m. This is in accordance with the theory of the beam transition to an infinite range in the far-field. The *x*- and *z*-errors are very similar due to the array symmetry, and they increase due to the expanding beamwidth in *x*- and *z*-directions. These simulations yield RMS errors of 78 cm and 24 cm for SISO and SIMO, respectively, and 14 cm for the SIMO *x*-*z* projection for the 1.5 m range as used in our measurement. These simulation results are a good match with our measurement results and therefore underpin our multipath and simulation model.

**Figure 11:**
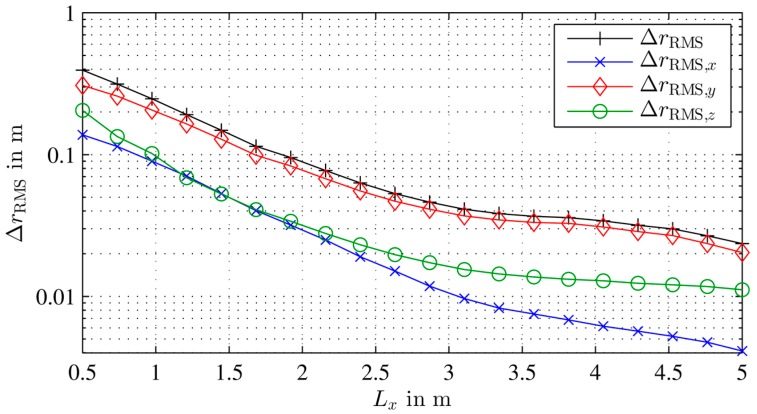
Simulation of the localization errors in *x*-, *y*- and *z*-directions for *K* = 6 dB. The RMS localization error decreases quickly as the edge length in the URA *x*-direction increases.

Final simulations demonstrate how it is possible to achieve a better localization accuracy in the measurement setup by changing size and element number of the URA. Figure 11 shows the overall RMS localization accuracy as well as the RMS accuracies in *x*-, *y*- and *z*-directions. The simulation uses K=10 dB and evaluates the errors for different aperture edge lengths in *x*-direction $L_{x}$ while maintaining an overall interelement spacing of 0.2 m and an edge length of 1 m in *z*-direction. By increasing the *x*-edge length from 1 to 3 m, the RMS error is reduced from 50 to 10 cm in the simulation setup. As expected, the *x*-accuracy profits from the now narrow beam in azimuth. Interestingly both, the *y*- and *z*-direction profit significantly from the bigger aperture in *x*-direction nearly as well.

**Figure 12:**
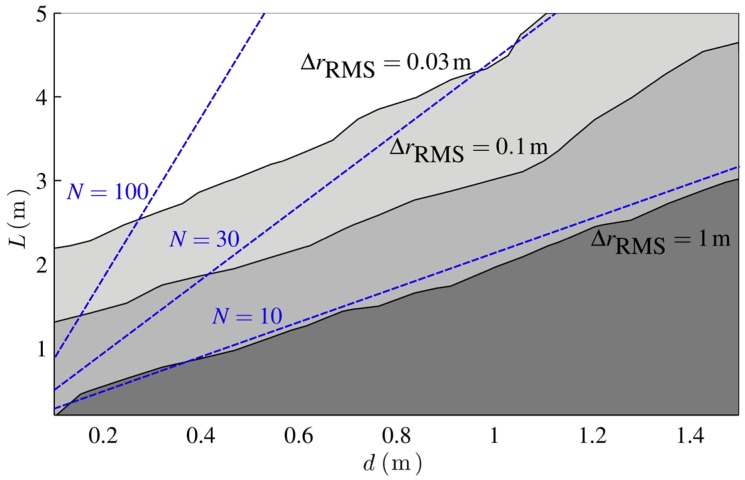
Simulation of the localization error for *K =* 10 dB for different URA edge lengths *L* and inter-element spacings *d*. Lines of constant element numbers *N* are indicated.

Figure 12 shows the simulated mean localization error for different combinations of the URA edge length *L* and interelement spacing *d* for K=10 dB and the SIMO transceiver mode. As expected, high localization accuracies are reached, for a low interelement spacing and a big aperture size. However, the constraints for the interelement spacing discussed previously do not impact the localization accuracy as much as in far-field systems. As the indicated lines of constant element number show, a big interelement spacing is compensated by a big aperture size, which even leads to more effectiveness in terms of necessary antenna elements per localization accuracy. Part of this effect can be explained with the spatial correlation of the multipath. A big aperture size leads to more uncorrelated multipath signals and the induced phase errors do not add up coherently.

## 10. Conclusions

The basic principles needed for the design of phase-based narrowband localization systems for backscatter tags have been discussed and evaluated by simulations and measurements. The simulations show which localization accuracies can be expected from a URA aperture for different localization algorithms, transceiver modes, noise and multipath conditions. Generally, it was shown that multipath dominates the localization error for UHF RFID systems. The simulations and measurements show that an error of 30 cm can be reached in a simple setup and how higher accuracies in the cm region can be achieved with increased aperture sizes, even under indoor multipath conditions. The methods we presented in this paper are easily combined with signal-strength-based methods or setups with moving tags to yield even higher accuracies. The huge design space of the proposed localization systems provides solutions to multiple applications of interest with different antenna geometries, reader architectures and signal processing algorithms.

## Abbreviations

The following abbreviations are used in this manuscript:

BF	Beamformer
DOA	Direction-of-arrival
EM	Electromagnetic
LOS	Line of sight
MIMO	Multiple input multiple output
MUSIC	Multiple signal classification
NFSL	Near-field source localization
RFID	Radio frequency identification
RSSI	Received signal strength indicator
RMS	Root mean square
S-MIMO	Singledimensional-MIMO
SIMO	Single Input Multiple Output
SISO	Single Input Single Output
UHF	Ultra high frequency
ULA	Uniform linear array
URA	Uniform rectangular array
